# Exploring the topological sources of robustness against invasion in biological and technological networks

**DOI:** 10.1038/srep20666

**Published:** 2016-02-10

**Authors:** Fernando Alcalde Cuesta, Pablo González Sequeiros, Álvaro Lozano Rojo

**Affiliations:** 1 GeoDynApp - ECSING Group (Spain); 2 Instituto de Matemáticas, Universidade de Santiago de Compostela, E-15782 Santiago de Compostela (Spain); 3 Departamento de Didáctica das Ciencias Experimentais, Facultade de Formación do Profesorado,Universidade de Santiago de Compostela, Avda. Ramón Ferreiro 10, E-27002 Lugo (Spain); 4 Centro Universitario de la Defensa Zaragoza, AGM, Ctra. Huesca s/n. E-50090 Zaragoza (Spain); 5 Instituto Universitario de Matemáticas y Aplicaciones, Universidad de Zaragoza (Spain)

## Abstract

For a network, the accomplishment of its functions despite perturbations is called robustness. Although this property has been extensively studied, in most cases, the network is modified by removing nodes. In our approach, it is no longer perturbed by site percolation, but evolves after site invasion. The process transforming resident/healthy nodes into invader/mutant/diseased nodes is described by the Moran model. We explore the sources of robustness (or its counterpart, the propensity to spread favourable innovations) of the US high-voltage power grid network, the Internet2 academic network, and the *C. elegans* connectome. We compare them to three modular and non-modular benchmark networks, and samples of one thousand random networks with the same degree distribution. It is found that, contrary to what happens with networks of small order, fixation probability and robustness are poorly correlated with most of standard statistics, but they depend strongly on the degree distribution. While community detection techniques are able to detect the existence of a central core in Internet2, they are not effective in detecting hierarchical structures whose topological complexity arises from the repetition of a few rules. Box counting dimension and Rent’s rule are applied to show a subtle trade-off between topological and wiring complexity.

A biological or technological system is *robust* if it continues to function despite perturbations. Different names have been used for this concept depending on the nature of the system, the particular feature to be considered robust, and the kind of change to be applied. *Scale-free networks*^1^ have been proposed as models of complex networks enjoying an unexpected degree of *robustness* or *error tolerance* when a fraction of nodes is removed at random. The Word-Wide-Web, the Internet and several social networks have been regarded as interesting examples by many authors. However, these real networks seem extremely vulnerable to targeted attacks on the highly connected nodes. Similar conclusions were stated for living systems like metabolic networks^2^ and PPI networks^3^ after computational removal of randomly chosen nodes. These results suggest an evolutionary selection of the topological structure of biological networks in both senses, global generating mechanisms that give rise to power laws, as local correlating degree connectivity and influence in the network.

To understand the paradoxical “robust yet fragile” nature of all these networks, other scenarios have been also explored. *Highly Optimised Tolerance (HOT) models*^4^ were proposed from the double perspective of the existence of technological and economical constraints limiting the network topology and the accomplishment of their functional objectives. Abilene, the backbone for the Internet2 academic network, was used to illustrate the performance of this kind of models after node removal^4^.

Our aim was to explore the topological sources of another kind of robustness of biological and technological networks. In our approach, such a network is no longer perturbed by site percolation, but it evolves after the attack of pathogens like viruses or prions. The evolutionary process transforming *healthy* (or *resident*) nodes into *diseased* (or *invader*) nodes is described by the Moran model on networks introduced by Lieberman *et al*.^5^. Morbidity depends on the relative *fitness* of the pathogen with respect to the immune answer of healthy nodes. More precisely, at the beginning, all nodes are healthy. Then, one node chosen at random becomes infected by the pathogen. At successive steps, a diseased or healthy node is chosen at random with probability proportional to the relative fitness 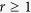 or 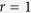. Next, a randomly chosen neighbour of the node is infected or cured. The *(average) fixation probability* is the probability that all the nodes of the network become infected. For a homogeneous or well-mixed networks this invasion process coincides with the classical Moran process^6^. In fact, *isothermal networks* has the same fixation probability that the homogeneous networks^5^. However, there are networks structures acting like evolutionary amplifiers favouring the disease spread across the network^5^,^7^,^8^,^9^.

In this context, we called *robustness against invasion* to a measure of the proximity of the network to the isothermal equilibrium. Here, we attempted to explore the topological sources of this kind of pathogen tolerance or its counterpart, the propensity to spread favourable innovations^10^. Therefore, motivated by the aforementioned works about usual robustness, we studied the robustness against invasion of the *US Power Grid (PG)*^11^ and *Internet2 (I2)* technological networks, and the neuronal network of the hermaphrodite nematode *Caenorhabditis elegans (CE)*^12^,^13^, see Data.

On the other hand, many researchers are interested in modularity and hierarchical modularity of technological and biological networks^14^,^15^,^16^,^17^,^18^,^19^,^20^, searching for adaptive, spatial, or economical constraints on their evolution^4^,^21^,^22^,^23^. According to them, modular architecture allows a faster adaptation to environmental changes, and their robustness is a major advantage when networks evolve under natural selection. Thus, we added a *hierarchical modular network (HR)*^20^ and a *random toy model (TW)*^21^ to contrast modularity and to test spatial aggregation propensity. Modular networks have the property of small-worldness^24^ characterised by a relative short average path length and a high clustering coefficient, favouring a low wiring cost. However, there are small-world networks that are not modular, so we also added the scale-free *Barabási-Albert model (BA)*^25^ to our study.

We computed the fixation probability using the Monte Carlo method on the associated Embedded Markov Chain, called the *EMC method*^7^. This allowed us to estimate the robustness against invasion of these networks. In an effort to understanding the structural causes of this robustness, we compared the fixation probability of the four deterministic networks PG, I2, CE and HR to that of samples of 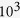 random networks having the same degree distribution.

## Results

### Mathematical model

Consider an undirected connected network 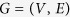 with node set 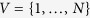 with no loops or multiple edges. Denote by 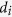 the degree of the node 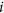. The *Moran process* on 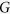 with fitness 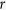 is the following Markov chain: start with a population 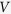 of 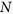 healthy nodes. Afterward, one single node 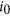 is chosen, with probability 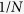, to become a diseased node. At successive steps one node 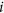 is selected at random with probability 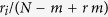, where 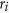 is 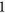 or 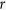 depending on whether 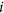 is healthy or diseased, and 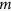 is the number of diseased nodes in that moment. Next, a neighbour of 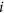, randomly chosen with probability 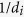, becomes healthy or diseased depending on whether 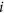 is healthy or diseased. The *fixation probability*, denoted 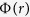, is the probability that the whole population becomes diseased at some time step. For more details, see Supplementary Information.

### Fixation function and robustness

**Figure 1:**
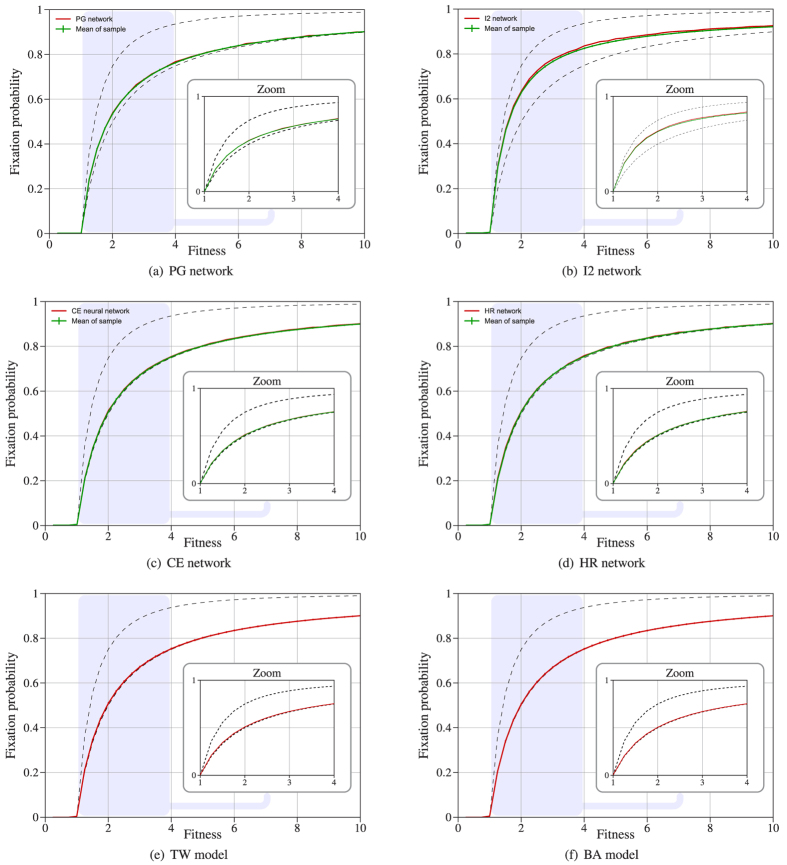
(**a**–**d**) Fixation probability functions for the US Power Grid, Internet2, *C. elegans* neuronal and hierarchical HR networks and asymptotically uniform samples of 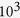 networks with the same degree distributions. We used 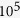 and 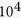 trials respectively for every fitness value 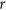 varying from 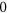 to 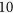 with step size of 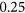. (**e**–**f**) Fixation probability functions for the Toy Worm and Barabási-Albert random models with samples of 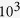 networks and 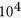 trials for the same values of 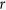.

The computation of the fixation probability leads to a system of 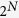 equations, see Supplementary Information. Hence, except where global or partial symmetries reduce the number of equations 5,7, analytically solving the system is infeasible if the order of the network is not a very small. Thus, we computed the fixation probability of the three real networks PG, I2 and CE and the deterministic hierarchical one HR applying the EMC method^7^ and using 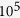 trials and fitness values 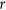 varying from 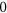 to 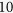 with step size of 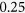. Each of these fixation functions is compared with the exact and asymptotic fixation functions 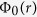 and 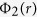 for equivalent sized complete and complete bipartite networks, which are given in Methods, Equations (2) and (3) respectively. Each of these fixation functions was also compared to the fixation function on average from a benchmark sample of 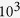 networks having the same degree distribution. Graphical representations of these fixation functions in Fig. 1 show a strong dependence on the degree distribution, which indeed increases for the most robust networks CE and HR. We repeated the computation for the random networks BA and TW, which also are robust networks.

To give a more accurate measure of the robustness of these networks, we introduce the *robustness against invasion*


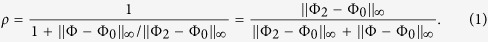


For details see Methods. We estimated the value of 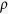 approximating 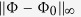 and 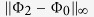 by the supremum of the differences 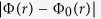 and 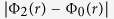 where 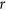 varies from 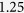 to 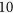 with steps of size 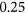. The data is shown in Table 1, which includes both average and standard deviation of 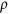 for random or randomised networks.

### Heterogeneity

**Figure 2:**
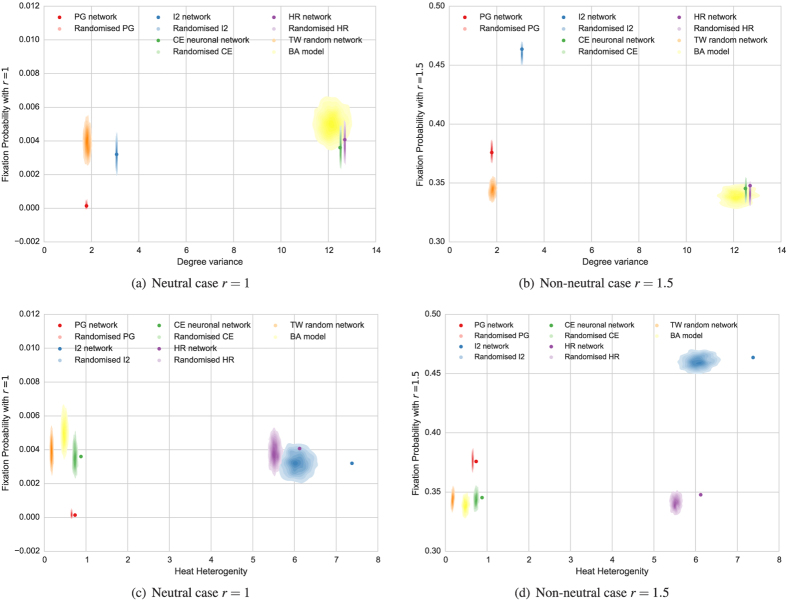
Comparing fixation probability in the neutral and non-neutral case with (**a**,**b**) variance of the degree distribution and, (**c**,**d**) heat heterogeneity^10^.

The notions of *heterogeneity*^26^,^27^ and *heat heterogeneity*^10^ (i.e. the variance of degree and temperature respectively) were proposed as strong indicators of the fixation probability for some small networks. We compared both heterogeneities with the fixation probability in the neutral case 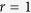 and in the non-neutral case 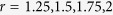. The most interesting cases 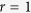 and 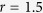 are shown in Fig. 2. The rest of the cases are similar to 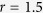, see Supplementary Information.

For the networks considered here, both heterogeneity and heat heterogeneity show a poor correlation with 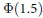, see Table 1 and Fig. 2. In fact, Kendall’s rank correlation coefficient values 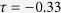 for 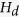 and 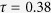 for 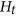. Figs 1 and 2 support the idea that robustness and fixation probability depend strongly on the degree distribution. So it is natural to study the correlation of the fixation probability in both neutral and non-neutral cases with other statistics directly related with the degree distribution such as *mean degree (*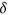), *degree median (*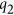), *degree skewness* and *degree kurtosis*, the network’s global scale such as *size (*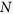), *edge number*, *diameter* and *temperature entropy (*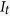), or the network’s topology such *clustering coefficient (*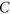), *transitivity ratio*, *average path length (*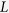), *power law exponent*, 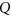
*-modularity*, 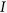
*-modularity* and *fractal dimension (*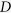). The values of these statistics are shown in Tables 1 and 2.

We concluded that 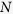 and 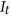 are the best correlated quantities with the fixation probability in the neutral case 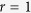, with 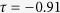 (having mutual Kendall’s 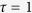). In the non-neutral case 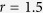 the most correlated statistics are the median degree with 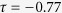, the mean degree with 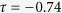, and the average path length 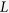 with 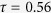, together with 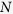 and 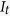 having 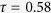, see Table 3. Nevertheless, at least for the networks considered here, robustness is moderately well correlated with the median degree with 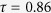, the mean degree 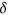 with 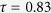 and the ratio 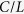 with 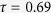, but it is rather poorly correlated with the other basic statistics. But it is reasonable to think that the correlations with the median and mean degree are biased by the nature of the considered networks, see Discussion.

### Modularity and hierarchical modularity

For many years, researchers have been interested in modularity and hierarchical modularity, searching for dynamic and evolutionary constraints that justify why biological and technological networks have a modular architecture. We were initially interested in two types of modularity. In the first one, the network splits into densely connected modules which are sparsely interconnected. The second one uses the idea that modular architecture reduces the information flow between modules.

**Figure 3:**
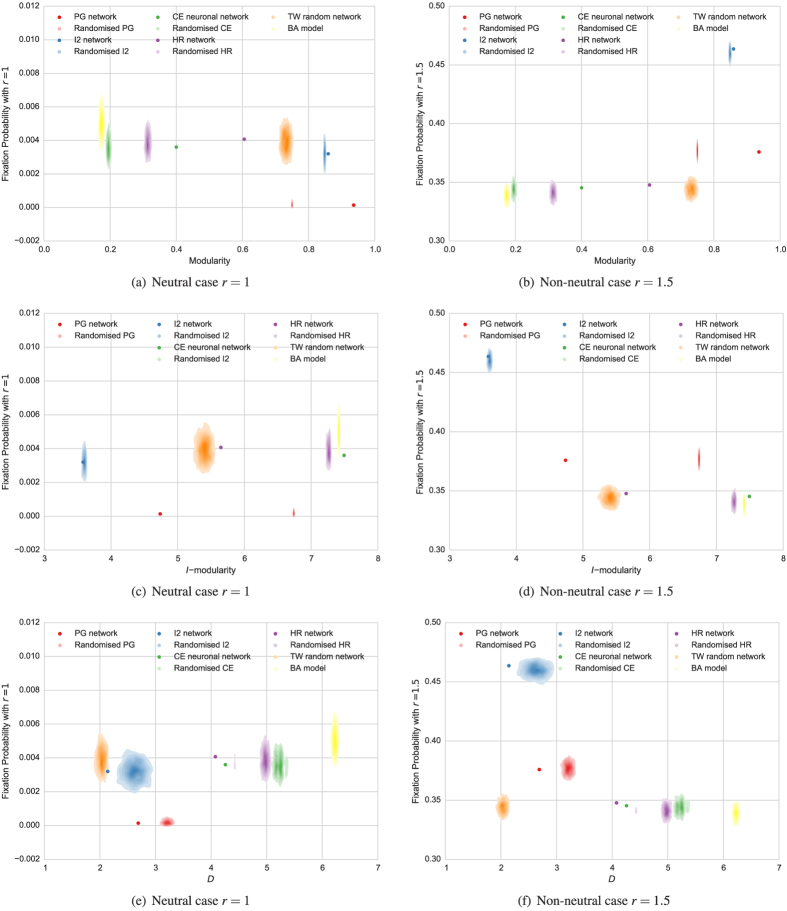
Comparing fixation probability in the neutral and non neutral cases with (**a**,**b**) 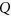-modularity, (**c**,**d**) 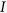-modularity and, (**e**,**f**) fractal dimension.

Thus, we computed the 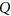-modularity^28^, using *Louvain method* proposed by Blondel *et al*.^29^. The results can be seen in Table 2, where 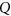 is compared with the robustness 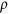 and the fixation probabilities 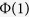 and 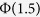, see also Fig. 3. For the networks considered here, the 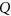-modularity has positive Kendall’s 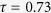 with respect to the fixation probability in the non-neutral case 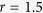 and negative 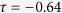 with respect to the robustness against invasion. The CE neuronal network shows a poor 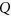-modularity, 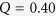, but significantly higher than that of the corresponding randomised network, 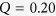. In general, 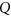-modularity is sensitive to the randomization process. The 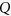-modularity of the hierarchical HR network is moderate, 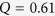, contrary to what happens with the technological networks PG and I2 with a high 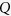-modularity and a central core or “hub subcomplex” in their structure.

**Figure 4:**
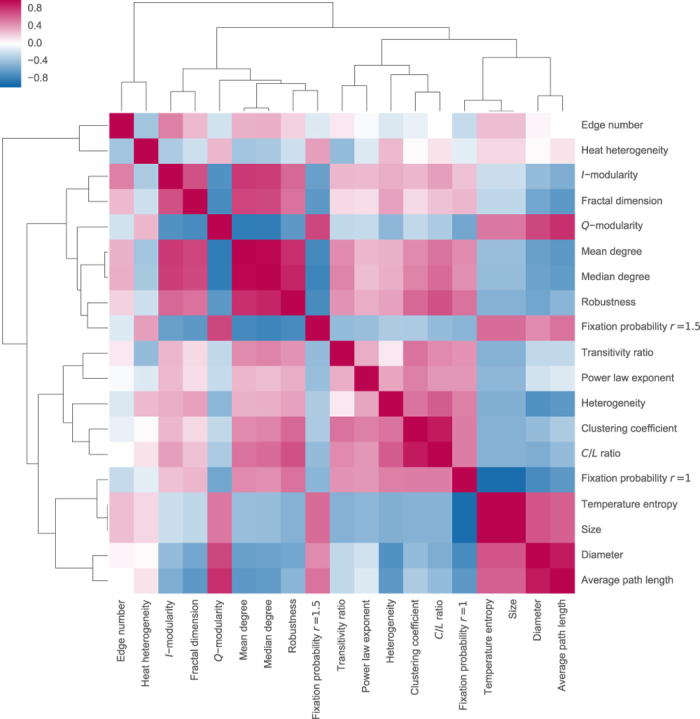
Comparing Kendall’s correlation coefficients.

We also adopted the point of view by Rosvall *et al*.^30^,^31^ replacing 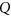 by the *Infomap code* or *minimum description length* 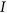, see Table 2 and Fig. 3. For the networks considered here the correlations of 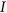 with the robustness 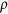 and the fixation probability 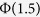 are a little worse than those of  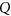. Both modularities has a mutual (negative) moderate correlation with 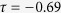, but they seem to have different nature according to the clustering in Fig. 4.

### Community structure

The idea that high 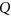-modularity is correlated with the existence of a central core or “hub subcomplex” was explicitly tested on the Internet2 network and the hierarchical HR networks of level 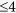. We used *Louvain algorithm*^29^ to detect the community structure of these networks. For I2 network, it produces a similar but not identical community structure to the actual one, suggesting a more suitable placement of some connectors, see Supplementary Information. As counterpoint to the robustness, the spreading of favourable innovations can be enhanced by this kind of structures^10^ that incorporate trade-offs between performance and available resources^4^. Thus, community detection techniques could allow us to optimise the accomplishment of the functional objectives of a network with the same resources. However, these techniques are not equally effective in detecting other hierarchical modular structures, like the HR network, more topologically complex but constructed by repeating a simple rule, see Supplementary Information for details. Consequently, we say that a network is *repetitive* if it is obtained from the repetition of a reduced number of deterministic or random rules that encode its topological and dynamical complexity.

### Topological complexity

Song *et al*. investigated the role of the fractal modular architecture in the robustness of some biological networks^32^,^33^. But the renormalisation mechanism that characterises this architecture continues to operate in the non-fractal case: there are models and examples of real networks showing a fractal behaviour in small scales, although they behave globally as small-worlds. We have seen that the BA model, and the CE and HR networks are robust small-worlds, while the PG and I2 networks are modular networks with central cores. From the point of view of both small-worldness (measured by the ratio 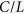) and modularity (measured by 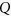), the robust TW random model appears to be at an intermediate position. It has no central core because of the construction itself.

Our computations of the fractal dimension 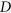 are consistent with the global picture sketched above, except for the random TW model which has the lowest fractal dimension. See Table 2. Like the over-representation of some motifs in both *C. elegans* neuronal^18^ and random TW^21^ networks, the robustness of both networks could be favoured by some kind of spatial aggregation. By its construction, TW has similar properties to the planar lattice, including its fractal dimension. There also might be spatial reasons for the properties of the *C. elegans* connectome, but obviously these cannot be the same as the given above.

Nevertheless, similarly to what happens with Rent’s rule^34^,^35^,^36^ to analyse internal communications in integrated circuits, fractality measures could be perturbed by the existence of two regions in the log-log plots. Namely, a *Region I* where the linear scaling show a fractal topology, and a *Region II* where the scaling is not linear but exponential. By restriction to Region I, we obtained new estimates, but the new data do not alter the picture above. See Supplementary Material for these results.

### Wiring complexity

In fact, Rent’s rule has been applied to some biological networks including *C. elegans* connectome^22^,^36^,^37^. Our estimates of Rent’s exponents somewhat differ from those obtained by these authors. This is due to the different ways in which boxes decompositions are constructed, using box counting instead of min-cut partition algorithms. Values for the random TW (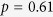), the PG (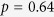) and the I2 (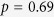) networks belong to the range of values that requires a cost-efficient wiring architecture for VLSI circuits. But the HR network has also a similar Rent’s exponent (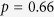) showing that a high topological complexity is compatible with a moderate wiring complexity. On the contrary, the BA model and the CE connectome exhibit high interconnection complexity with Rent’s exponents 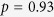 and 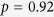 respectively. In all cases, randomisation process increases Rent’s exponents as well as the fractal dimension. Now, a combination of repetitiveness with some randomness, which is missing in the HR network, could justify a gain of topological and wiring complexity without affecting the robustness of network like the CE connectome.

Finally, Fig. 4 shows a global picture of the correlations. The hierarchical clustering of different statistics (with respect to the taxicab metric) is illustrated with a dendrogram. Most notable is the clustering of the modularity measures, in especial 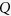 and 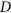, around the robustness and the fixation probability in the non-neutral case 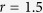.

## Discussion

We computed the fixation functions for US Power Grid, Internet2 and *C. elegans* neuronal networks and asymptotically uniform samples of 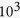 randomly constructed networks with the same degree distributions. We completed these calculations with the fixation probability functions of four other benchmark networks: the hierarchical network constructed by Ravasz *et al*.^20^, the corresponding randomised sample of 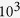 networks with the same degree distribution, the random toy model constructed by Artzy-Randrup *et al*.^21^ and the Barabási-Albert model^25^, both with the same sample size as above. Moreover, we introduced the *robustness against invasion* to measure the proximity of a network to the isothermal equilibrium, that is, the equivalence of the network to a homogeneous population from the point of view of the Moran process. This quantity is interpreted as pathogens tolerance.

We distinguished two different groups: a first group of robust small-worlds formed by the BA model and the CE and HR networks with the corresponding randomised networks, and a second group of modular networks with central cores formed by PG and I2 with their corresponding randomised benchmarks. Regarding small-worldness and modularity, the TW model appears to be in an intermediate position. In the paper, we attempted to explore the topological sources of the robustness against invasion.

Initially, heterogeneity^26^,^27^ and heat heterogeneity^10^, defined as the variances of the degree and temperature distributions, were proposed as statistics well correlated with the fixation probability on some networks of small order. But neither heterogeneity, nor heat heterogeneity seem to have a determining effect on the fixation probability of these technological and biological large networks. In fact, we have shown that there is a strong dependence on the whole degree distribution. Consequently, it is not surprising that specific statistical correlations are not too high.

In the neutral case 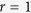, we proved that the size and the temperature entropy^8^ are equally very well (negatively) correlated with the fixation probability 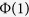. In the non-neutral case 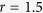, the median degree and the mean degree are also (negatively) well correlated to the fixation probability 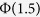, which now gives a measure of the propensity to spread favourable innovations^10^. With respect to the robustness 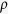, the ratio 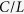 between the average path length 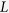 and the clustering coefficient 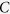 is also moderately correlated. But as evidenced by our own numerical experiments (to be published elsewhere), correlations with respect the median and mean degree are much lower on networks of small order.

Small-worldness of the *C. elegans* neuronal network was evidenced by other authors. In some cases, they used the same model as we did to describe the CE connectome^15^. In other cases, the model is similar, but not exactly the same^12^,^22^,^23^, even if the data have been obtained at the same source^13^. Our estimations for 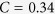 and 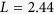 coincide with those given by Kim *et al*.^15^, except that we rounded up to 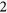 decimal places.

To precise the global picture of the biological and technological networks considered here, we also estimated the 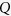-modularity, the 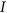-modularity and the fractal dimension of these networks. As explained in Methods, the 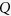-modularity was calculated by using the Louvain method proposed by Blondel *et al*.^29^. In fact, the same method was already used by Basset *et al*.^22^ to estimate 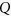 for the CE connectome obtaining the same low value 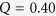. On the other hand, using an older algorithm^38^, Pan *et al*.^23^ gave a slightly lower estimate 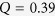 where all the numbers are still rounded up to 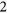 decimal places. In fact, this authors make another estimate assuming that communities in CE connectome correspond to ganglia where 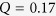. The same approach (but using a different algorithm) was applied by Kim *et al*.^15^ obtaining a value 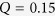. Note however that these results could be biased by the edge/link swapping algorithm to randomise CE connectome. Regarding fractality, the use of the box counting method for networks with low diameter represents the main limitation, because these networks are rapidly covered by a single box. This applies to the BA model and the CE and HR networks, as well as their randomised benchmarks. In the particular case of the CE connectome, Bassett *et al*.^22^ used a standard partitioning algorithm to estimate what they call *topological fractal dimension* 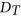. Explicitly, they obtained the value 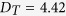 which is higher than the box-counting dimension 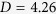 and the box-counting dimension 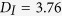 in restriction to the Region I. Nevertheless, their results using the box-counting method coincide with ours, both included in the corresponding Supplementary Informations.

Our results are also consistent with previous observations correlating 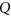-modularity with the existence of a central core or “hub subcomplex” in the network, although one must be careful about interpreting the 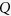-modularity values obtained here: high for the technological networks PG and I2, both modular with central core, medium for HR and TW, and low for BA and CE. The idea that a high 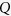-modularity is correlated with the existence of this kind of core has been tested by describing explicitly the community structure of the Internet2 network and the HR networks of level 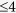. While community detection techniques are able to identify the central core of Internet2, and even suggest improvements in its efficiency, they are not equally effective in detecting other hierarchical modular structures where topological complexity comes from the repetition of a single or finite set of rules. The BA model and the HR network are examples of such repetitive networks. BA shows low 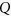-modularity and high fractal dimension whereas the 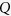-modularity and the fractal dimension of HR are moderate and high respectively. Moreover, as we proved, the randomisation processes on these examples always increases the fractal dimension, which measures their topological complexity.

When networks are decomposed into boxes in order to compute their fractal dimension, it is natural to ask for the relationship between the number of interconnections and the box size. We applied Rent’s rule to estimate the interconnection or wiring complexity of the network. Although this power law was initially formulated for VSLI circuits^34^, a number of authors used Rent’s rule to analyse other biological and technological networks^22^,^36^,^37^. Using the box counting method, we privileged topological aspects against geometrical ones, even if those are certainly interesting. Based on our own method to determine Region II (and also Region III introduced by Stroobandt^39^), we obtained estimates of Rent’s exponents that slightly differ from those obtained by these authors, but allowed us to draw a reasonable schema: the BA model and the CE connectome have a high wiring complexity, while the other networks have moderate values corresponding to cost-efficient wiring architectures. Moreover, randomisation processes increases Rent’s exponents, a property which was also observed by Reda^36^ for other biological and technological networks. The HR network shows that a high topological complexity and a moderate wiring complexity are compatible for a network.

Values for Kendall’s coefficient correlating robustness and fixation probability in the non-neutral case 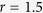 with 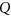-modularity, 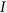-modularity and fractal dimension, see Table 3, suggest a certain correlation with a suitable combination of topological and wiring complexity. However, as we have seen, HR and TW are robust networks with a similar wiring complexity, but a very different topological complexity. The reason for the low fractal dimension of TW resides in its own geometrical construction favouring a sort of spatial aggregation, which is similar to that of a planar lattice. On the contrary, a combination of repetitiveness with some randomness, not present in HR, could justify a gain of topological and wiring complexity without affecting the robustness against invasion of a network like the CE connectome.

Summing up, from the comparison of the *C. elegans* neuronal network with the technological networks PG and I2 and the benchmark models TW and BA emerges the idea of a subtle trade-off between high complexity and low cost. In fact, according to a number of authors^14^,^16^,^17^,^19^,^22^ and based on their observations, this phenomenon could have been favoured by the evolution. Now, we have a triple challenge: firstly, analyse in more detail the influence of the degree distribution on the robustness, secondly, give a measure of the repetitiveness for networks of any order, and thirdly, quantify its effect on the robustness.

## Methods

### Robustness

For a homogeneous network 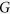 of size 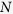, the Moran process on 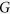 reduces to the classical Moran process where all nodes have the same fixation probability


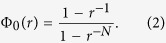


Let 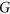 be a *weight-balanced network*, that is, the weights of entering edges 
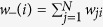
 and leaving edges 
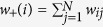
 are equal for any node 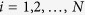. Then, according to the Circulation Theorem^5^, the number of elements of each state performs a biased random walk on the integer interval 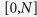 with forward bias 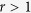 and absorbing states 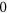 and 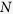. Therefore, the fixation probability is the same as that of the homogeneous network of size 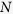. In fact, if 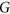 is undirected, the weight of entering edges of a node 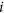 can be interpreted as the *temperature* 
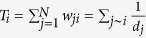
, where 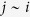 means that 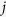 is a neighbour of 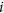 and 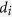 is the number of neighbours of 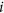. Hence, an undirected weight-balanced network is *isothermal* because 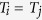 for all nodes 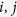.

On the other hand, the fixation probability of complete bipartite networks 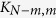 converges to the same limit that the fixation probability


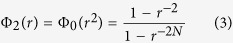


of the Moran process with fitness 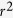 as 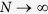
7. In other words, these structures are *evolutionary amplifiers* favouring advantageous invaders.

Let 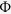 be the fixation function of a network of size 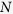. In order to measure the distance between 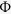 and 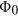, we used the norm 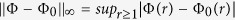 and the ratio 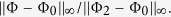 The *robustness against invasion* of a network is the quotient


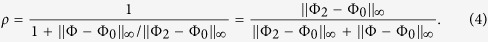


Hence, any isothermal network has robustness against invasion 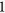.

### Networks data

The *US Power Grid (PG) network* is the high-voltage power grid in the Western States in the USA^11^. The nodes are generators, transformers, or substations and the edges are high-voltage transmission lines. Originally used by Watts and Strogatz^24^, this undirected network has 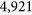 nodes and 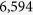 edges.

The *Internet2 (I2) network* assembles data from Internet2 community, available now through the Global Research Network Operations Center (GlobalNOC) at Indiana University^40^, which were collected in April 2013. First, we considered the list of active Internet2 Connectors at October 2012. It is an essential part of the Internet2 Combined Infrastructure Topology as described at September 2010. Secondly, we added the list of active Internet2 Primary Participants at April 2013 according to the GlobalNOC website^40^. For more details on the lists of connectors and primary participants, see Supplementary Information. Thus, we obtain a network with 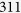 nodes and 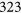 edges. The initial Abilene network (which was replaced by Internet2 in October 2007) was previously analised by Doyle *et al*.^4^.

The *C. elegans neuronal (CE) network* used here incorporates original data from^41^ and updates based upon later work^42^,^12^. This version of *C. elegans* connectome has 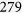 somatic neurones, 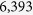 chemical synapses, 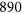 gap junctions, and 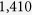 neuromuscular junctions^13^. According to our aim, we do not distinguish directionality of connections in this network that combines undirected gap junctions with directed chemical synapses, ignoring neuromuscular junctions and synaptic multiplicities. Thus, all the unidirectional connections between two different neurones will be replaced by bidirectional ones leading to a total of 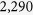 edges connecting 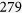 nodes. Since neurones RIBL/R and VA08 have auto-connections, we restrict our attention to 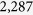 connections, cf. refs 12,15,22,23. There were efforts on the description of prionic diffusion within the brain^43^ and the construction of replication models^44^, which are consistent with the Moran process. An analysis of the human brain cluster structure detected by Gallos *et al*.^45^ using fMRI techniques (where modular clusters belong to a region of 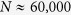 activated voxels) would require additional computational effort.

**Figure 5:**
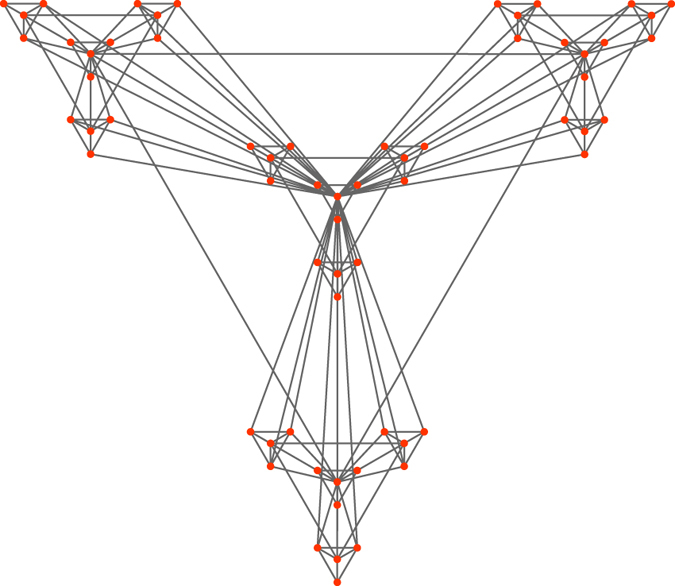
Hierarchical network of level 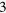 constructed by Ravasz *et al*.^20^.

The *Hierarchical (HR) network* was constructed by Ravasz *et al*.^20^ as an example of hierarchical modular organisation, see Fig. 5. The one used here has four different levels leading to a total of 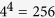 nodes and 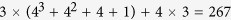 edges.

The *Toy Worm (TW) network*^21^ is a random network sampled as follows: it consists of 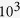 networks of order 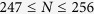 which were obtained from a square of 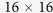 points in the integer lattice. Let 
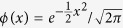
 be the density function of the standard normal distribution. Two different nodes 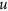 and 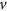 in the square are connected by an edge with probability 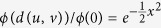 depending on the Euclidian distance 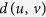. The distribution of the number of connected components and the order frequencies of the maximal ones are showed in Supplemetary Information. The mean order is 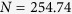 with a standard deviation of 1.26.

Finally, the *Barabási-Albert (BA) model* is another random network constructed using the preferential attachment process^25^. Here, we sampled 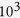 networks of order 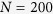 starting from 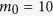 initial nodes and attaching each new node 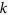 to 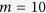 nodes with probability 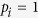 or 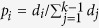 for 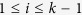, depending on whether 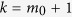 or 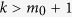. We needed 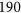 iterations to complete each network having 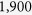 edges.

### Random networks with prescribed degree distribution and fixation probability computation

The problem of generating random networks with a prescribed degree distribution was discussed by many authors^46^,^47^,^48^,^49^. In our case, random network generation was done in NetworkX^50^ using the Markov chain scheme proposed by Gkantsidis *et al*.^47^.

We compared the robustness of the three real networks PG, I2 and CE and the hierarchical deterministic one HR to that of their respective benchmark families of random networks having the same degree distribution. Each family consists of an asymptotically uniform sample of 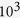 networks generated by using the ‘swap’ algorithm^47^, where 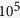 true double-edge swaps were done on each real or deterministic network.

On the other hand, to estimate the average fixation probability for each element of the sample we used the EMC method^7^. We computed the fixation probability function 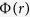 using sequences of 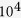 trials for each fitness value 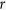 varying from 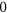 to 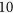 with step size of 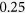 for each of the 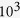 networks in the sample. For the other random networks 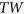 and 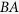, we did the same simulation from the initial sample of 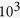 networks. The fixation probability function of the original networks PG, I2, CE and HR was computed using 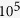 trials. Finally, we estimated its robustness against invasion (4).

### Statistics

We analysed several statistical properties of the four networks PG, I2, CE and HR and their respective randomised benchmarks, as well as those of the two random networks BA and TW. Firstly, for each real or deterministic network, we considered some standard measures such as *mean degree*, *degree median*, *degree variance*, *degree skewness* and *degree kurtosis* related with the degree distribution. We also considered the *heat heterogeneity*^10^ defined as the variance of the temperature distribution 
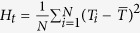
, where 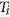 is the temperature of node 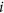 and 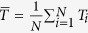 is the mean temperature. As above, for the other random networks, we computed these measures on average from the initial sample of 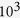 networks. Secondly, for any undirected connected network 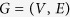, in addition to the *size* 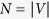 and the *edge number* 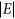, we consider other global measures such as the *diameter* 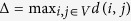, where 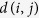 is the length of the shortest path between the nodes *i* and *j*, and the *temperature entropy^8^* 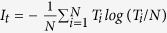. Finally, we studied small-word properties of 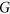 starting by the *clustering coefficient* 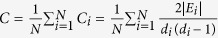, where 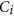 is the density of the induced sub-network 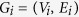 consisting of the set 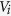 of neighbours of 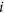 and the set 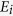 of edges between neighbours of 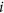. The *transitivity ratio* is a measure of the network transitivity defined as the ratio between the number of nodes in triangles and the triplets of nodes connected by two edges. The *average path length* of 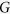 is given by 
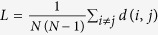
. *Power law exponents* were calculated using the *power law* Python package^51^ to adequately fit the tail of each degree distribution by a power law with respect to a optimal minimal value 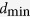.

### Rank correlation measures

A rank correlation coefficient is a measure of monotone dependence between two numerical random variables when ranked according to their values. Giving a set 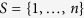 consisting of 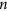 individuals, two quantitive properties of these individuals are represented by two vectors 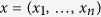 and 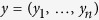. To each pair of individuals 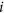 and 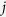, we associate antisymmetric 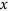-score 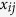 and 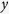-score 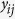. A rank correlation coefficient^52^ is defined by


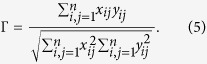


When 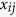 is the difference between the ranks of 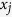 and 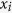, *Spearman’s rank correlation coefficient* is obtained. Here, we considered *Kendall’s* 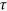 derived from (5) by choosing


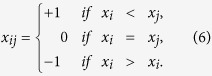


In our case, each deterministic, random or randomised network is treated as a single network in order to state monotone dependence between two statistics (on average for random or randomised networks). For ordered sets with 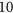 elements, the upper critical value at a significance level of 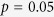 is 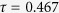. Statistical computations and graphics were done using the Python scientific stack^53^,^54^,^55^,^56^.

### Modularity and hierarchical modularity

A quantity called 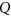
*-modularity*^28^,^38^ was introduced by Girvan and Newman to measure the decomposability of a network into modules. Given a partition 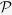 of the vertex set 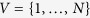 of an undirected connected network 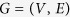, the *modularity of* 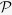 is equal to 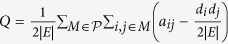, where 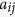 is the entry of the adjacency matrix corresponding to two nodes 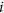 and 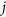 in some module 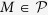. Several algorithms were proposed to detected the modular structure of the network by finding partitions with the largest value of 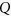. See a comparative analysis in ref. 38. Recently, methods to study hierarchical modularity were introduced starting from the idea of decomposing modules into submodules, which in turn are decomposed into sub-submodules, and so on. Here, we used the *Louvain method*^29^, which takes advantage of the hierarchical structure of the network to accelerate the optimisation of 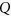.

However, as discussed in the comparative analysis by Lancichinetti *et al*.^57^, there are other approaches to identify community structures. We also used the *Infomap* method by Rosvall *et al*.^30^,^31^. The modular structure of the network is now achieved by optimising a function, called the *minimum description length* 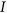, which gives account of the balance between information flow and data compression^30^,^31^.

### Fractal dimension

An alternative definition of modular network was introduced by Song *et al*.^32^,^33^ representing modules by boxes of different length scales. Any network 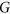 admits a decomposition into disjoint *boxes of size* 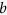, i.e. finite sets of nodes of diameter 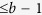. The fractality of 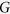 can be formulated as an invariance property by the renormalisation flow, which replaces each box with a “renormalised” node. The *fractal dimension* 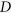 of 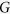 can be estimated by two equivalent ways: as the exponent of a power law for the number of boxes of size 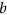



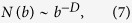


or as the exponent of a power law for the average number of nodes 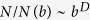 in a box of size 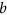 when 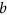 varies from 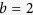 to the first integer 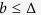
such that 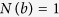.

In general, for scale-free networks, it is convenient to use the first method based on power law (7) to find the fractal scaling, although several algorithms can be used to calculate the fractal dimensions, each of them with its pros and cons^58^,^59^. Here, we implemented the greedy colouring algorithm proposed by Song *et al*.^58^ according to the description given by Locci *et al*.^59^. The fractal dimension of PG, I2, CE and HR networks was estimated using the ordinary least squares regression on the data gathered from the above algorithm. For each random and randomised network, each of the 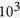 sample elements was partitioned and then 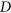 was estimated both computing fractal dimension on average (with the corresponding standard deviations) and fitting a single line to the whole data set. In all cases, standard errors in the fit of the power law (on average in random ones) were also computed and included in Table 2. Naturally, errors decrease as the diameter of network increase. But there is another source of possible error in the estimation of 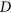 related with random choices in the construction of the partitions. However, other authors found low values of the standard deviation^60^ which have been corroborate by our own numerical experiments. So we have chosen at random a single partition for each of 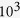 sample elements.

## Additional Information

**How to cite this article**: Alcalde Cuesta, F. *et al*. Exploring the topological sources of robustness against invasion in biological and technological networks. *Sci. Rep.* **6**, 20666; doi: 10.1038/srep20666 (2016).

## Supplementary Material

Supplementary Information

## Figures and Tables

**Table 1:** Robustness and fixation probability in the non-neutral case *r* = 1.5 compared with mean degree δ, heterogeneity *H*_*d*_, heat heterogeneity *H*_*t*_ and some small-worldness measures (on average for asymptotically uniform samples of 10^3^ networks in random networks).

***Network***	***ρ***	**Φ(1.5)**	***δ***	***H***_***d***_	***H***_***t***_	***C***	***L***	***C*****/*****L***
BA	0.954 ± 0.010	0.339 ± 0.005	19.00	12.14 ± 0.37	0.48 ± 0.04	0.18 ± 0.01	2.06 ± 0.01	0.089 ± 0.004
CE	0.948	0.345	16.39	12.49	0.88	0.34	2.44	0.138
HR	0.946	0.348	6.87	12.69	6.12	0.59	2.41	0.246
HR random	0.942 ± 0.010	0.341 ± 0.005	6.87	12.69	5.53 ± 0.07	0.25 ± 0.01	2.33 ± 0.01	0.108 ± 0.003
CE random	0.942 ± 0.011	0.344 ± 0.005	16.39	12.49	0.74 ± 0.03	0.14 ± 0.00	2.27 ± 0.01	0.062 ± 0.002
TW	0.940 ± 0.012	0.344 ± 0.005	4.75 ± 0.15	1.83 ± 0.09	0.18 ± 0.02	0.24 ± 0.02	6.51 ± 0.18	0.037 ± 0.003
PG	0.855	0.376	2.67	1.79	0.74	0.08	18.99	0.004
PG random	0.845 ± 0.010	0.377 ± 0.005	2.67	1.79	0.65 ± 0.01	0.00 ± 0.00	8.71 ± 0.03	0.000 ± 0.000
I2 random	0.649 ± 0.007	0.460 ± 0.005	2.08	3.06	6.07 ± 0.23	0.00 ± 0.00	5.63 ± 0.26	0.000 ± 0.000
I2	0.639	0.464	2.08	3.06	7.38	0.00	8.26	0.000

Networks are sorted by robustness.

**Table 2:** Robustness and fixation probabilities compared with *Q*-modularity, *I*-modularity and fractal dimension, on average for asymptotically uniform samples of 10^3^ networks in random networks.

***Network***	***ρ***	**Φ(1)**	**Φ(1.5)**	***Q***	***I***	***D***	***std err***	***R***	***D*****′**
BA	0.954 ± 0.010	0.005 ± 0.001	0.339 ± 0.005	0.17 ± 0.00	7.41 ± 0.01	6.22 ± 0.08	0.01	0.962	6.22
CE	0.948	0.004	0.345	0.40	7.49	4.26	0.18	0.995	4.26
HR	0.946	0.004	0.348	0.61	5.65	4.08	1.40	0.810	4.08
HR random	0.942 ± 0.010	0.004 ± 0.000	0.341 ± 0.005	0.31 ± 0.01	7.26 ± 0.02	4.95 ± 0.13	0.02	0.945	4.92
CE random	0.942 ± 0.011	0.004 ± 0.001	0.344 ± 0.005	0.20 ± 0.00	7.81 ± 0.00	5.20 ± 0.15	0.01	0.984	5.18
TW	0.940 ± 0.012	0.004 ± 0.001	0.344 ± 0.005	0.73 ± 0.01	5.40 ± 0.07	2.03 ± 0.06	0.00	0.979	2.02
PG	0.855	0.000	0.376	0.94	4.74	2.69	0.06	0.978	2.69
PG random	0.845 ± 0.010	0.000 ± 0.000	0.377 ± 0.005	0.75 ± 0.00	6.74 ± 0.01	3.21 ± 0.06	0.01	0.910	3.21
I2 random	0.649 ± 0.007	0.003 ± 0.001	0.460 ± 0.005	0.85 ± 0.00	3.60 ± 0.02	2.63 ± 0.15	0.00	0.976	2.58
I2	0.639	0.003	0.464	0.86	3.58	2.14	0.09	0.970	2.14

For fractal dimension we include the coefficient of determination *R* in the fit of the power law (7). For each random or randomised network, all the networks in the sample are considered at once, fitting the whole data set by a single regression line of slope *D*′. Differences between fractal dimension on average and exponent in a global fit are less than 0.05.

**Table 3:** Kendall’s rank correlation coefficient for robustness and fixation probability in the neutral and non-neutral cases (*r* = 1 and *r* = 1.5) with respect some statistics sorted by their absolute values with respect to the robustness.

	***q***_**2**_	***δ***	***C***/***L***	***Q***	***I***	***C***	***D***	***N***	***I***_***t***_	***L***
*ρ*	0.86	0.83	0.69	−0.64	0.60	0.60	−0.56	−0.49	−0.49	−0.47
Φ(1)	0.44	0.46	0.51	−0.56	0.24	0.51	0.29	−0.91	−0.91	−0.64
Φ(1.5)	−0.77	−0.74	−0.42	0.73	−0.60	−0.33	−0.64	0.58	0.58	0.56
