# Supplementary material for: Exploring the topological sources of robustness against invasion in biological and technological networks
Source: Sci Rep. 2016 Feb 10;6:20666. doi: 10.1038/srep20666 (PMC4748249; doi:10.1038/srep20666)
Supplement: Supplementary Information [file srep20666-s1.pdf]

Exploring the topological sources of robustness to invasion in biological and technological networks
Supplementary information

Fernando Alcalde Cuesta[1,2], Pablo González Sequeiros[1,3], and Álvaro Lozano Rojo[1,4,5]

[1]GeoDynApp - ECSING Group (Spain)
[2]Facultade de Matemáticas, Universidade de Santiago de Compostela, E-15782 Santiago de Compostela (Spain)
[3]Departamento de Didáctica das Ciencias Experimentais, Facultade de Formación do Profesorado, Universidade de Santiago de Compostela, Avda. Ramón Ferreiro 10, E-27002 Lugo (Spain)
[4]Centro Universitario de la Defensa, Academia General Militar, Ctra. Huesca s/n. E-50090 Zaragoza (Spain)
[5]Instituto Universitario de Matemáticas y Aplicaciones, Universidad de Zaragoza (Spain)

S1 Moran process on networks

Evolutionary dynamics has been classically studied for homogeneous finite populations. The *classical Moran model*[1] describes the change of gene frequency by random drift on a population of finite size N. This model has many variants, but here we start with a population of resident individuals. At the beginning, one single individual is randomly chosen to become mutant or invader. At each time step, one individual is chosen at random for replication occupying the place of another individual chosen at random to be eliminated. To model natural selection, it suffices to assume that mutant or invader individuals have relative fitness $r > 1$ as compared to the resident ones whose fitness is 1.

Liberman et al.[2] introduced a generalisation of this model by arranging the population on a directed or undirected network, see also.[3,4] Here, we considered an undirected connected network $G = (V,E)$ with node set $V = \{1,\dots,N\}$, which has no loops or multiple edges. The *Moran process* on G is a Markov chain X_n whose states are the sets of nodes S inhabited by mutant (or invader) individuals at each time step n. The reproductive (or invasive) advantage is measured by the fitness $r \geq 1$ and the transition probabilities of the Markov chain are defined from the stochastic matrix $W = (w_{ij})$ given by $w_{ij} = 1/d_i$ if $(i,j) \in E$ and $w_{ij} = 0$ otherwise, where d_i is the degree of the node i. More precisely, the transition probability between two states S and S' is given by

$$P_{S,S'} = \begin{cases} \dfrac{r\sum_{i\in S} w_{ij}}{r\sum_{i\in S}\sum_{j\in V} w_{ij} + \sum_{i\in V\setminus S}\sum_{j\in V} w_{ij}} & \text{if } S'\setminus S = \{j\}, \\ \dfrac{\sum_{i\in V\setminus S} w_{ij}}{r\sum_{i\in S}\sum_{j\in V} w_{ij} + \sum_{i\in V\setminus S}\sum_{j\in V} w_{ij}} & \text{if } S\setminus S' = \{j\}, \\ \dfrac{r\sum_{i,j\in S} w_{ij} + \sum_{i,j\in V\setminus S} w_{ij}}{r\sum_{i\in S}\sum_{j\in V} w_{ij} + \sum_{i\in V\setminus S}\sum_{j\in V} w_{ij}} & \text{if } S = S', \\ 0 & \text{otherwise,} \end{cases} \tag{1}$$

where $r\sum_{i\in S}\sum_{j\in V} w_{ij} + \sum_{i\in V\setminus S}\sum_{j\in V} w_{ij} = r|S| + N - |S| = N + (r-1)|S|$ is the sum of the reproductive (or invasive) weights of the mutant (or invaders) and resident individuals. The *fixation probability* of any set S inhabited by mutant or invaders individuals

$$\Phi_S(r) = \mathbb{P}[\exists n \geq 0 : X_n = V | X_0 = S]$$

is obtained as solution of the linear equation

$$P\Phi(r) = \Phi(r), \tag{2}$$

as for the classical Moran process on a homogeneous finite population. Since G is undirected, the only recurrent states are $S = \emptyset$ and $S = V$, and then it is well known that (2) has a unique solution, see [5, Sec. III.7]. In this context, the

(average) fixation probability is

$$\Phi(r) = \frac{1}{N}\sum_{i=1}^{N}\Phi_{\{i\}}(r).$$

Mutant and residents nodes have been called diseased and healthy nodes when we applied the Moran model to biological networks evolving after the attack of a pathogen, but failures in power grids can be also modeled by this stochastics process.

S2 Internet2 network connectivity lists

The *Internet2 network* assembles data from Internet2 community, available through the Global Research Network Operations Center (GlobalNOC) at Indiana University,[6] which were collected in April 2013. The list of active Internet2 Connectors at October 2012 is given in Table S1. It is an essential part of the Internet2 Combined Infrastructure Topology as described at September 2010. Secondly, the list of active Internet2 Primary Participants at April 2013 is given in Table S2. For more recent lists of connectors and primary participants, see also Tables S1 and S2.

Table S1. List of active Internet2 Connectors at October 2012 and May 2015 according to
http://noc.net.internet2.edu/i2network/maps--documentation/peers/active-connectors.html

October 2012	May 2015
3ROX/Drexel = Three Rivers Optical Exchange/Drexel University	3ROX/Drexel
	CAAREN = Capital Area Advanced Research and Education Network
CENIC = Corporation for Education Network Initiatives in California	CENIC
CIC OmniPoP = Committee on Institutional Cooperation	CIC OmniPoP
FLR/SoX = The Southern Crossroads	FLR = Florida Lambda Rail<br>SOX = Southern Crossroads
GPN = Great Plains Network	GPN
Indiana GigaPoP	Indiana GigaPoP
KyRON = Kentucky Regional Optical Network	KyRON
LEARN = Lonestar Education and Research Network	LEARN
LONI = Louisiana Optical Network Initiative	LONI
MAGPI = Mid-Atlantic Gigapop in Philadelphia for Internet2	MAGPI
MARIA = Mid-Atlantic Research Infrastructure Alliance	MARIA
MAX = Mid-Atlantic Crossroads	MAX
MCNC/C-Light = North Carolina Research and Education Network	MCNC/C-Light
MissiON = Mississippi Optical Network	MissiON
MREN = Metropolitan Research and Education Network	MREN
NOX = The Northern Crossroads	NOX
NYSERNet = NYSERNet Inc.	NYSERNet
OARnet = Ohio Technology Consortium	Ohio Academic Resources Network
Oregon Gigapop	Oregon Gigapop
PNG = Pacific Northwest Gigapop	PNG
	Sun Corridor Network
University of Memphis	University of Memphis
UEN = Utah Education Network	Utah/Montana

Table S2. List of active Internet2 Primary Participants according to
http://noc.net.internet2.edu/i2network/maps-documentation/peers/active-participants.html

Number of Participants at April 2013	Inactive participants at May 2015	New active participants at May 2015
253	University of Medicine and Dentistry of New Jersey	Brown University<br>NYSERNet, Inc<br>The College of William and Mary

S3 Distribution of the number of connected components and order frequencies in the random Toy Worm network

The *Toy Worm (TW) network* is a random network constructed by Artzy-Randrup et al.[7] Here, it consists of 10^3 networks of order $247 \leq N \leq 256$ which were obtained from a square of 16×16 points in the integer lattice. Let $\varphi(x) = e^{-\frac{1}{2}x^2}/\sqrt{2\pi}$ be the density function of the standard normal distribution. Two different nodes u and v in the square are connected by an edge with probability $\varphi(d(u,v))/\varphi(0) = e^{-\frac{1}{2}x^2}$ depending on the Euclidian distance $d(u,v)$. The distribution of the number of connected components and the order frequencies of the maximal ones are showed in Table S3. The mean order is $N = 254.74$ with a standard deviation of 1.26.

Table S3. Distribution of the number of connected components and order frequencies for the maximal one for the sample in the random Toy Worm network.

#	Frequency
1	324
2	349
3	204
4	94
5	21
6	6
7	2

Order	Frequency
247	1
248	1
250	3
251	12
252	39
253	99
254	191
255	330
256	324

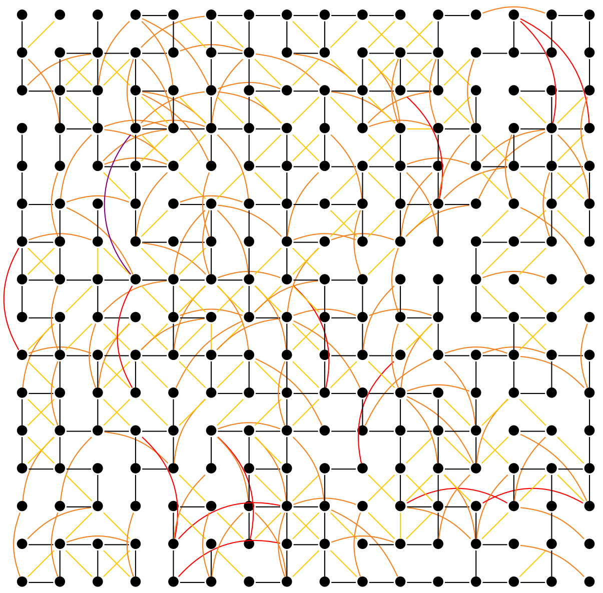

Figure S1. A single state of the Toy Worm network

S4 Hetereogeneity in the non-neutral case

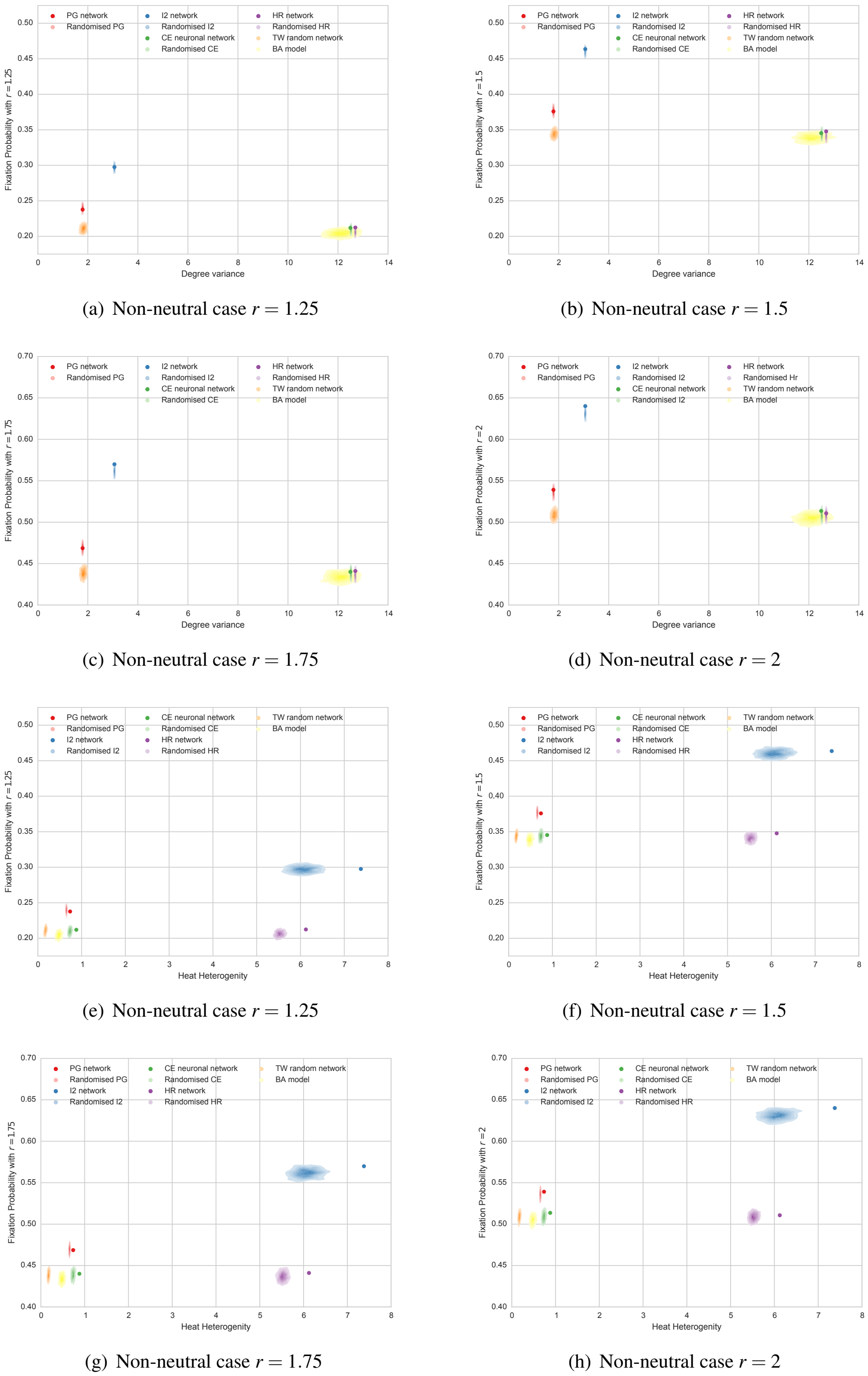

(a) Non-neutral case $r = 1.25$
(b) Non-neutral case $r = 1.5$
(c) Non-neutral case $r = 1.75$
(d) Non-neutral case $r = 2$
(e) Non-neutral case $r = 1.25$
(f) Non-neutral case $r = 1.5$
(g) Non-neutral case $r = 1.75$
(h) Non-neutral case $r = 2$

Figure S2. Comparing fixation probability with (a)-(d) variance of the degree distribution and (e)-(h) heat heterogeneity[8] in the non-neutral case.

S5 Modularity measures in the non-neutral case

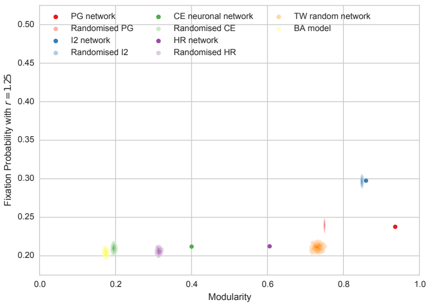

(a) Non-neutral case $r = 1.25$

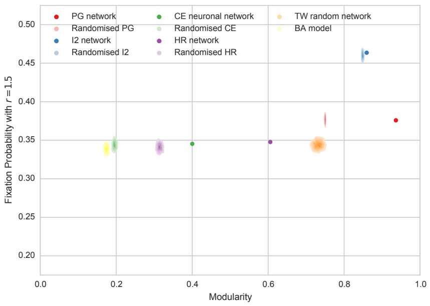

(b) Non-neutral case $r = 1.5$

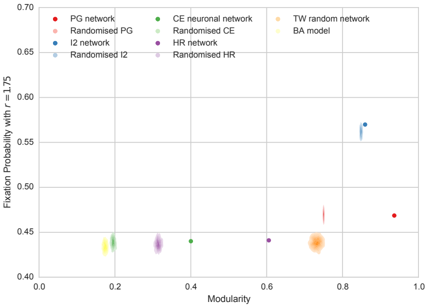

(c) Non-neutral case $r = 1.75$

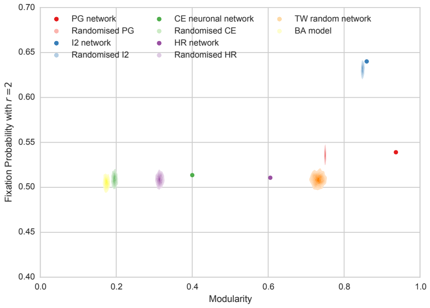

(d) Non-neutral case $r = 2$

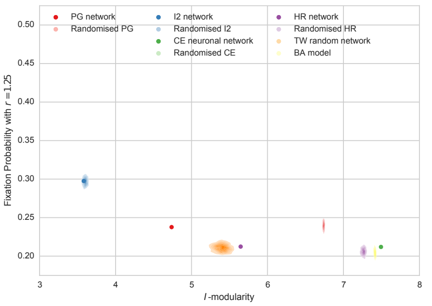

(e) Non-neutral case $r = 1.25$

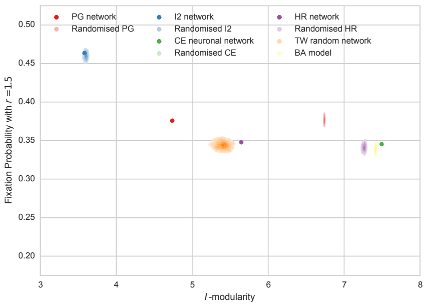

(f) Non-neutral case $r = 1.5$

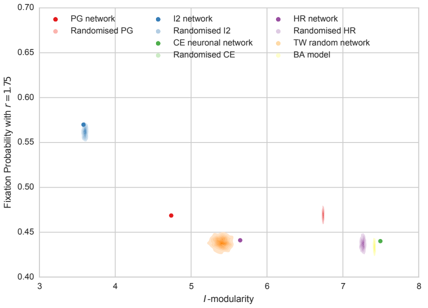

(g) Non-neutral case $r = 1.75$

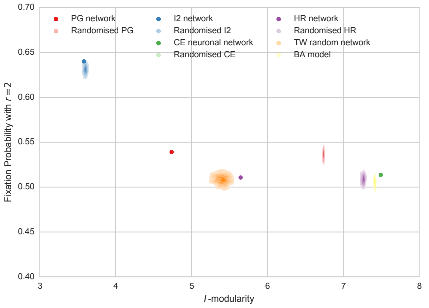

(h) Non-neutral case $r = 2$

Figure S3. Comparing fixation probability with (a)-(d) Q-modularity and (e)-(h) I-modularity in the non-neutral case.

S6 The community structure of Internet2

We used *Louvain algorithm*[9] to detect the community structure of the Internet2 academic network:

Table S4. The central core and the community structure of Internet2. Next to the name, the degree of each hub is shown in the table.

Central core	Modules
CENIC - 15	CENIC = Corporation for Education Network Initiatives in California - 15<br>Tionesta node<br>Sacramento node<br>San Francisco node<br>Sunnyvale node<br>San Luis Obispo node<br>California Institute of Technology<br>California Polytechnic State University-San Luis Obispo<br>California State University, Fullerton<br>California State University, Office of the Chancellor<br>California State University-East Bay<br>Claremont Colleges<br>Complete Genomics Inc.<br>HP<br>Jet Propulsion Lab<br>Naval Postgraduate School<br>Pepperdine University<br>Stanford University<br>UCAR/NCAR (University Corporation for Atmospheric Research)<br>University of California, Office of the President<br>University of California-Berkeley<br>University of California-Davis<br>University of California-Irvine<br>University of California-Los Angeles<br>University of California-Riverside<br>University of California-San Francisco<br>University of California-Santa Barbara<br>University of California-Santa Cruz<br>University of Nevada-Las Vegas<br>University of Southern California
CIC OmniPoP - 16	CIC OmniPoP = Committee on Institutional Cooperation OmniPoP - 16<br>St. Louis node<br>Detroit node<br>Marquette University<br>Mayo Medical School College of Medicine<br>Michigan State University<br>Missouri University of Science and Technology<br>North Dakota State University Main Campus<br>South Dakota School of Mines and Technology<br>South Dakota State University<br>University of Illinois at Urbana-Champaign<br>University of Michigan-Ann Arbor<br>University of Minnesota-Twin Cities<br>University of Missouri - Columbia<br>University of Missouri - Saint Louis<br>University of North Dakota Main Campus<br>University of South Dakota<br>University of Toledo<br>University of Wisconsin-Madison<br>University of Wisconsin-Milwaukee<br>Wayne State University
FLR/SoX - 14	MissiON = Mississippi Optical Network - 5<br>FLR/SoX = The Southern Crossroads - 14<br>Auburn University<br>Centers for Disease Control and Prevention<br>Emory University<br>Georgia Institute of Technology<br>Georgia State University<br>Jackson State University<br>Medical University of South Carolina<br>Mississippi State University<br>Oak Ridge National Laboratory<br>University of Georgia<br>University of Mississippi<br>University of South Carolina - Columbia<br>University of Southern Mississippi<br>University of Tennessee
GPN - 8	GPN = Great Plains Network - 8<br>Boise node<br>Rieth node<br>Tulsa node<br>Arkansas State University<br>Idaho State University<br>Iowa State University<br>Kansas State University<br>Oklahoma State University- Main Campus<br>The University of Montana<br>University of Arkansas at Little Rock<br>University of Arkansas for Medical Sciences<br>University of Arkansas Main Campus<br>University of Idaho<br>University of Iowa<br>University of Kansas Main Campus<br>University of Missouri - Kansas City<br>University of Oklahoma Norman Campus<br>University of Tulsa<br>Wichita State University

Indiana GigaPoP - 8	Indiana GigaPoP - 8<br>University of Memphis - 2<br>KyRON = Kentucky Regional Optical Network - 4<br>Nashville connector<br>Birmigham node<br>C-SPAN<br>Indiana University<br>Purdue University Main Campus<br>Saint Louis University<br>University of Alabama at Birmingham<br>University of Alabama in Huntsville<br>University of Alabama, The<br>University of Kentucky<br>University of Louisville<br>University of Memphis<br>University of Notre Dame<br>Vanderbilt University<br>Washington University in St. Louis
LEARN - 10	LEARN = Lonestar Education and Research Network - 10<br>Dallas connector<br>Baylor College of Medicine<br>Baylor University<br>Rice University<br>Southern Methodist University<br>Stephen F. Austin State University<br>Texas A&M University<br>Texas Christian University<br>Texas Tech University<br>University of Houston<br>University of North Texas<br>University of Texas at Arlington<br>University of Texas at Austin<br>University of Texas at Dallas<br>University of Texas Medical Branch At Galveston<br>University of Texas Southwestern Medical Center at Dallas
LONI - 5	LONI = Louisiana Optical Network Initiative - 5<br>Mobile node<br>Tallahassee node<br>Jacksonville connector<br>Florida Agricultural and Mechanical University<br>Florida Atlantic University<br>Florida International University<br>Florida State University<br>Louisiana State University<br>New World Symphony<br>Tulane University<br>University of Central Florida University of Florida<br>University of Miami<br>University of New Orleans<br>University of Puerto Rico<br>University of South Florida
MAGPI - 13	MAGPI = Mid-Atlantic Gigapop in Philadelphia for Internet2 - 13<br>Lehigh University<br>New Jersey Institute of Technology<br>Princeton University<br>Rutgers, The State University of New Jersey<br>Temple University<br>The Children's Hospital of Philadelphia<br>The Philadelphia Orchestra Association<br>University of Delaware<br>University of Medicine and Dentistry of New Jersey<br>University of Pennsylvania<br>JSTOR
MAX - 25	MAX = Mid-Atlantic Crossroads - 25<br>American University<br>Catholic University of America<br>Federal Highway Administration, Turner-Fairbank Highway Research Center<br>George Washington University (The)<br>Georgetown University<br>Howard Hughes Medical Institute<br>Johns Hopkins University<br>Laboratory for Telecommunications Sciences (LTS)<br>Library of Congress<br>Morgan State University<br>NASA Goddard Space Flight Center<br>National Archives and Records Administration<br>National Institutes of Health<br>National Library of Medicine<br>National Science Foundation<br>NIST (National Institute of Standards and Technology)<br>NOAA (National Oceanic & Atmospheric Administration, Washington, D.C.)<br>Northrop Grumman Corporation<br>United States Department of Agriculture, Agricultural Research Services (USDA)<br>University of Maryland Baltimore<br>University of Maryland Baltimore County<br>University of Maryland College Park
MCNC/C-Light - 9	MCNC/C-Light = North Carolina Research and Education Network - 9<br>MARIA = Mid-Atlantic Research Infrastructure Alliance - 7<br>Charlotte connector<br>Clemson University<br>Duke University<br>East Carolina University<br>George Mason University<br>North Carolina State University<br>Old Dominion University<br>Red Hat, Inc.<br>University of North Carolina at Chapel Hill<br>University of North Carolina At Greensboro<br>University of Virginia<br>Virginia Commonwealth University<br>Virginia Polytechnic Institute and State University<br>Wake Forest University

MREN - 9	MREN = Metropolitan Research and Education Network - 9<br>Bradley University<br>DePaul University<br>Loyola University Chicago<br>Northern Illinois University<br>Northwestern University<br>Southern Illinois University Carbondale<br>University of Chicago<br>University of Illinois at Chicago
NOX - 22	NOX = The Northern Crossroads - 22<br>Albany node<br>New York City connector<br>Berklee College of Music<br>Boston College<br>Boston University<br>Brandeis University<br>Columbia University<br>Dartmouth College<br>EBSCO Industries, Inc<br>EBSCO Information Services<br>ESPN, Inc<br>Harvard University<br>IBM Corporation<br>Manhattan School of Music<br>Massachusetts Institute of Technology (MIT)<br>New York University<br>Northeastern University<br>Rensselaer Polytechnic Institute<br>Stony Brook University, State University of New York<br>The City University of New York<br>Tufts University<br>University At Albany, State University of New York<br>University of Connecticut<br>University of Maine<br>University of Massachusetts<br>University of New Hampshire<br>University of Rhode Island<br>University of Vermont<br>Worcester Polytechnic Institute<br>Yale University<br>Yeshiva University<br>JSTOR
OARnet - 13	OARnet = Ohio Technology Consortium - 13<br>3ROX/Drexel = Three Rivers Optical Exchange - 6<br>NYSERNet = NYSERNet Inc. - 8<br>Buffalo connector<br>Binghamton University<br>Carnegie Mellon University<br>Case Western Reserve University<br>Cleveland Institute of Music<br>Cornell University<br>Kent State University Main Campus<br>Marshall University<br>Miami University-Oxford<br>Ohio University -Main Campus<br>Penn State (The Pennsylvania State University)<br>Rochester Institute of Technology<br>Syracuse University<br>The Ohio State University<br>University At Buffalo, The State University of New York<br>University of Akron, Main Campus, The<br>University of Cincinnati Main Campus<br>University of Pittsburgh, Pittsburgh Campus<br>University of Rochester<br>West Virginia University<br>Wright State University
PNG - 10	PNG = Pacific Northwest Gigapop - 10<br>OG = Oregon Gigapop - 5<br>Microsoft Research<br>Montana State University - Bozeman<br>Oregon Health & Science University<br>Oregon State University<br>Portland State University<br>Portland State University<br>University of Alaska Fairbanks<br>University of Hawaii At Manoa<br>University of Oregon<br>University of Washington<br>Washington State University
UEN - 5	UEN = Utah Education Network - 5<br>Reno node<br>Eureka node<br>Denver connector<br>Rawlins node<br>Ogden node<br>Heartwell node<br>Omaha node<br>Brigham Young University<br>Colorado School of Mines<br>Colorado State University<br>Desert Research Institute<br>JSTOR<br>United States Antarctic Program (USAP)<br>United States Department of Commerce Boulder Labs<br>University of Colorado at Boulder<br>University of Colorado Denver<br>University of Denver<br>University of Nebraska - Lincoln<br>University of Nevada, Reno<br>University of Utah<br>University of Wyoming<br>Utah State University

33 El Paso node - 6	San Diego<br>Phoenix node<br>Tucson node<br>El Paso node<br>Valentine node<br>Sanderson node<br>San Antonio node<br>Rancho de la Fe node<br>Albuquerque node<br>Raton node<br>Arizona State University<br>New Mexico State University -Main Campus<br>Northern Arizona University<br>San Diego State University<br>University of Arizona<br>University of California-San Diego<br>University of New Mexico Main Campus<br>University of Texas at El Paso<br>University of Texas at San Antonio

S7 The community structure of the hierarchical HR networks

We also used *Louvain algorithm*[9] to detect the community structure of the hierarchical HR networks from level 0 to level 4.

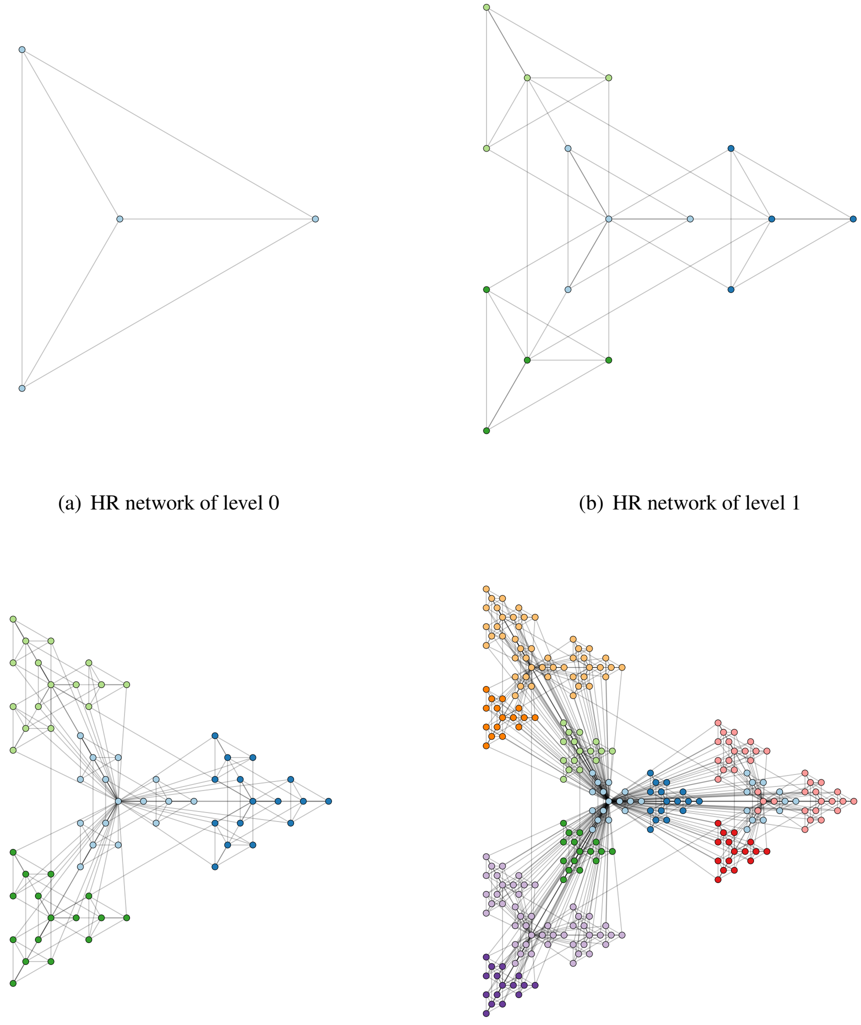

(a) HR network of level 0

(b) HR network of level 1

(c) HR network of level 2

(d) HR network of level 3

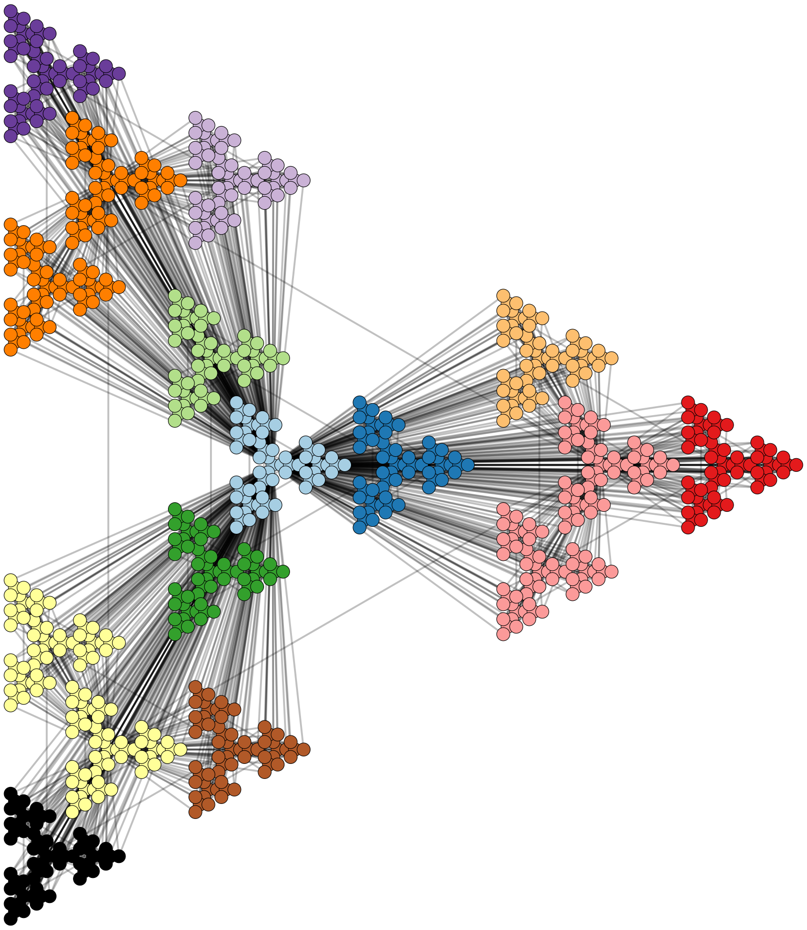

(e) HR network of level 4

Figure S4. The community structure of the hierarchical HR networks from level 0 to level 4 given by the Louvain algorithm.[9]

S8 Box-counting fractal dimension

We used the box counting method to estimate the fractal dimension of all the networks considered here. Since this fractality measure is perturbed by the existence of Region II, where the scaling is not linear but exponential, we also give another estimate in restriction to Region I.

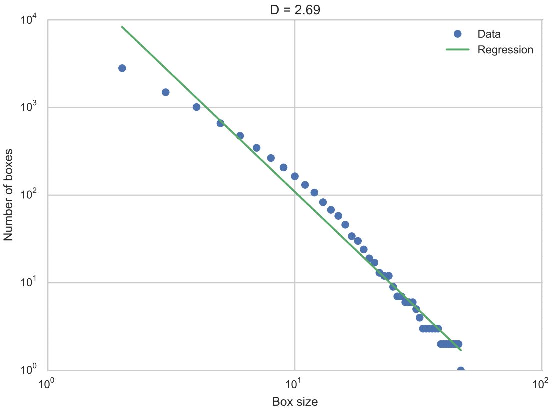

(a) US Power Grid network

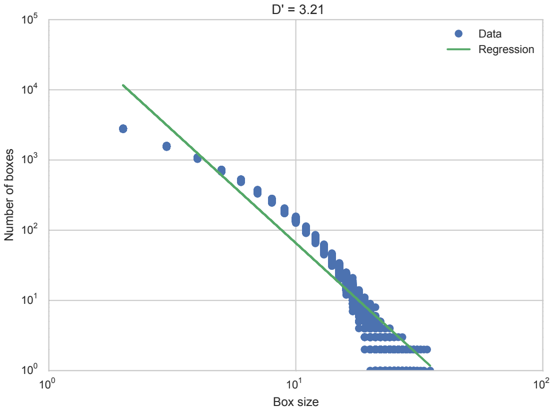

(b) US Power Grid randomised network

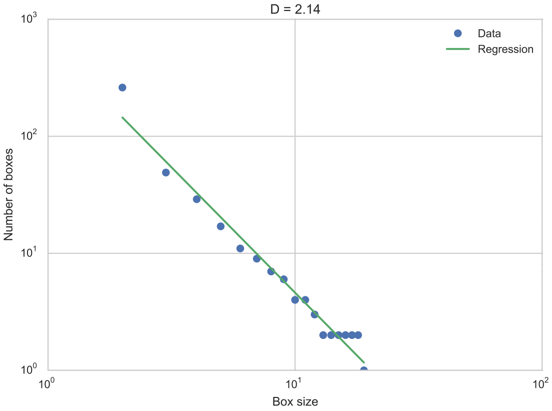

(c) Internet2 network

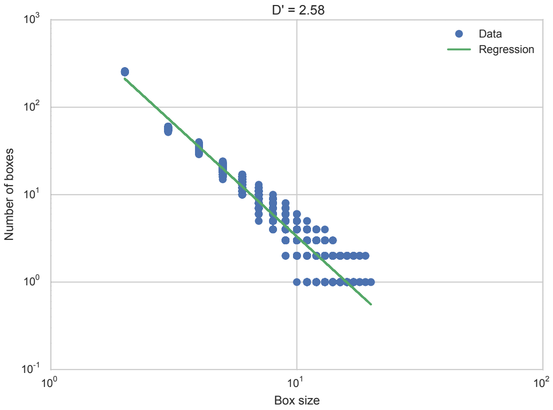

(d) Internet2 randomised network

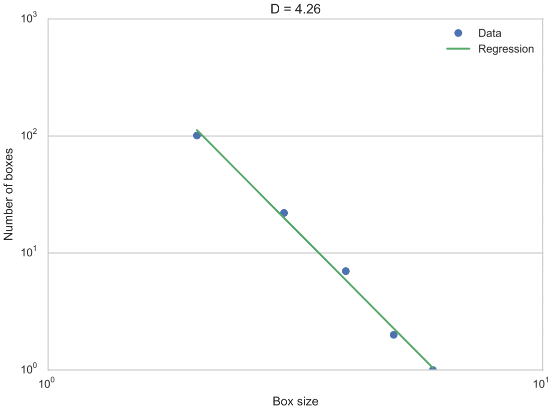

(e) *C. Elegans* connectome

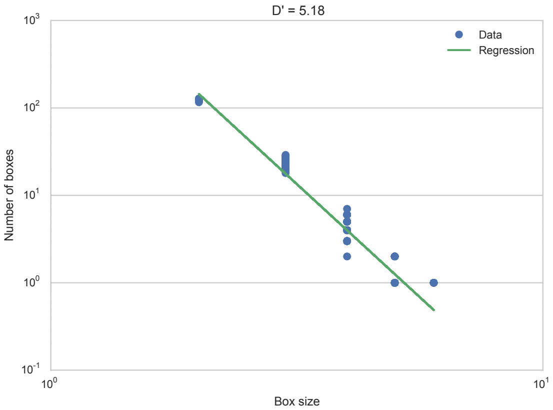

(f) *C. Elegans* randomised connectome

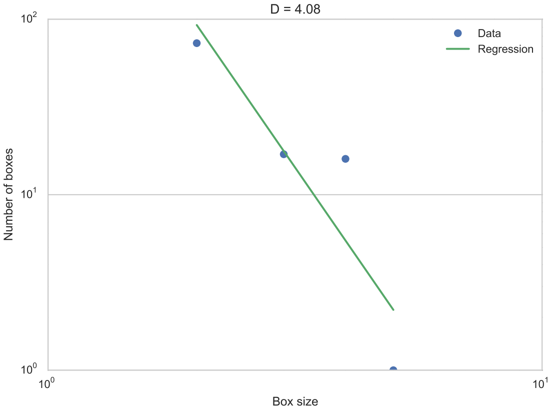

(g) Hierarchical network

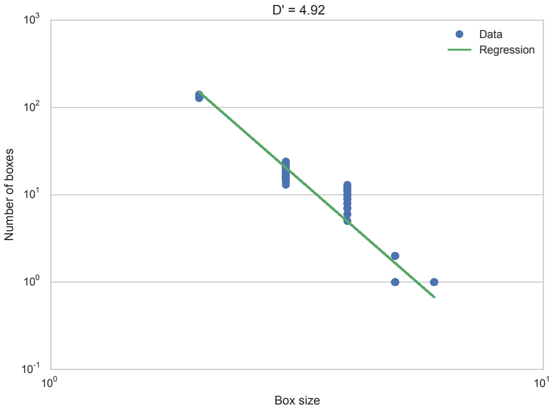

(h) Hierarchical randomized network

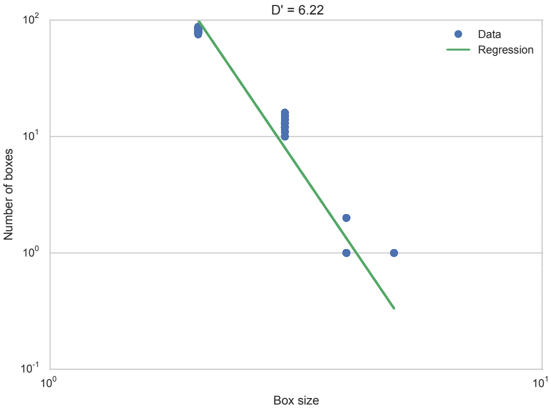

(i) Barabási-Albert model

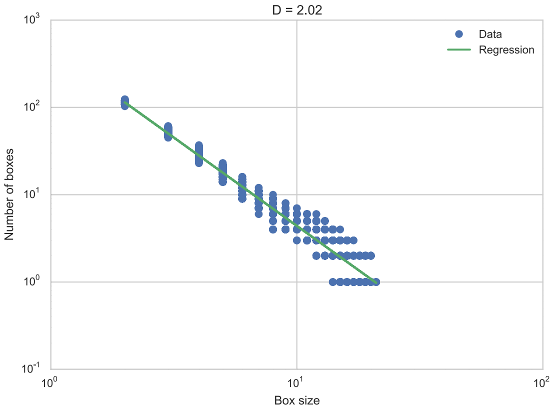

(j) Toy Worm random network

Figure S5. The fractal dimension D has been estimated as the slope of the log-log regression line for the number of boxes $N(b)$ of size b. In all cases, the greedy colouring algorithm proposed by Song et al.[10] has been implemented to decompose each network into boxes of all the possible sizes from $b = 2$ to the first integer $b \leq \Delta$ such that $N(b) = 1$. For random or randomised networks, we have considered all the values of $N(b)$ for the 10^3 elements of the sample at the same time, although only 50 values are depicted in each figure.

Table S5. Robustness ρ and fractal dimension D are compared with the slope D_I of the log-log regression line in restriction to Region I that gives a new estimate of the fractal dimension.

	ρ	D	D_I
BA	0.954 ± 0.010	6.22	6.23
CE	0.948	4.26	3.76
HR	0.946	4.08	3.59
HR random	0.942 ± 0.010	4.95	4.80
CE random	0.942 ± 0.011	5.20	4.12
TW	0.941 ± 0.012	2.03	2.06
PG	0.855	2.69	2.53
PG random	0.845 ± 0.010	3.21	2.09
I2 random	0.649 ± 0.007	2.63	2.53
I2	0.639	2.14	2.40

S9 Fractal dimension in the non-neutral case

In the paper, we compared the fractal dimension with the fixation probability in the neutral and non-neutral case $r = 0$ and $r = 1.5$. This comparaison is completed by adding the cases $r = 1.25, 1.75, 2$.

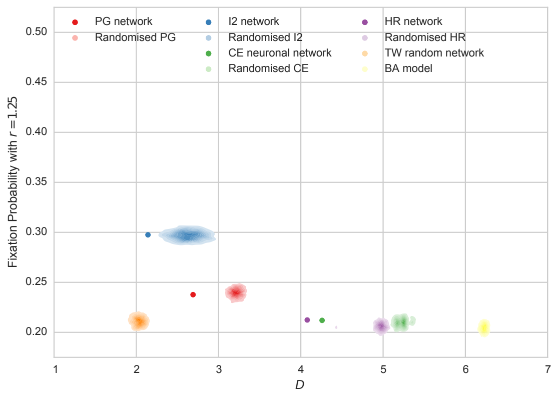

(a) Non-neutral case $r = 1.25$

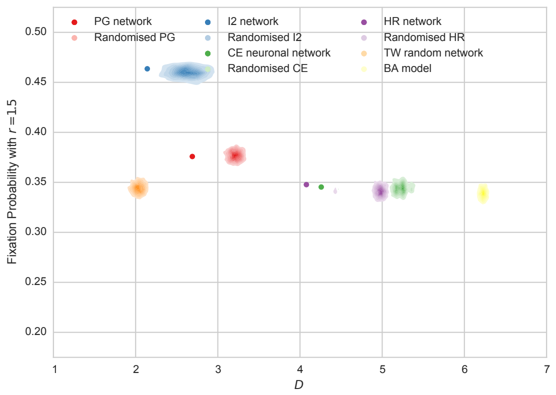

(b) Non-neutral case $r = 1.5$

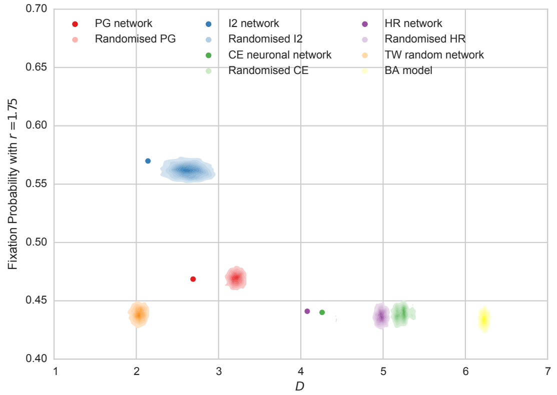

(c) Non-neutral case $r = 1.75$

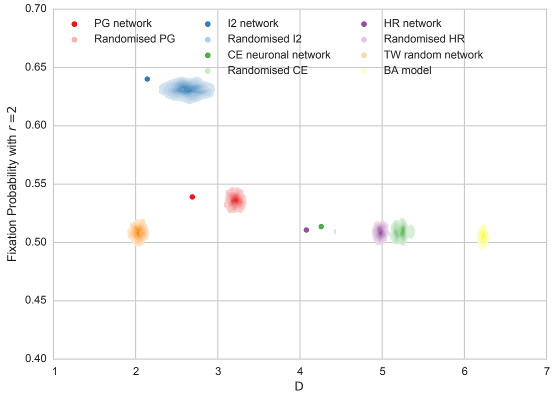

(d) Non-neutral case $r = 2$

Figure S6. Comparing fixation probability with fractal dimension in the non-neutral case.

S10 Kendall's rank correlation coefficients

We analysed correlation between robustness, fixation probability (in the neutral and non-neutral cases $r = 1, 1.5, 2$) and a number of statistics, see Figure S7. Graph order and temperature entropy are the best correlated with the fixation probability in the neutral case $r = 1$. In the non-neutral $r = 1.5$, these statistics are the median degree q_2, the mean degree δ and the average path length L, together with modularity measures. Nevertheless, robustness is moderately well correlated with q_2, δ and C/L, but it is rather poorly correlated with the other basic statistics. Q-modularity is positive correlated with respect to the fixation probability in the non-neutral case $r = 1.5$ and negative with respect to the robustness to invasion. Correlations for I-modularity and fractal dimension have slightly lower absolute values.

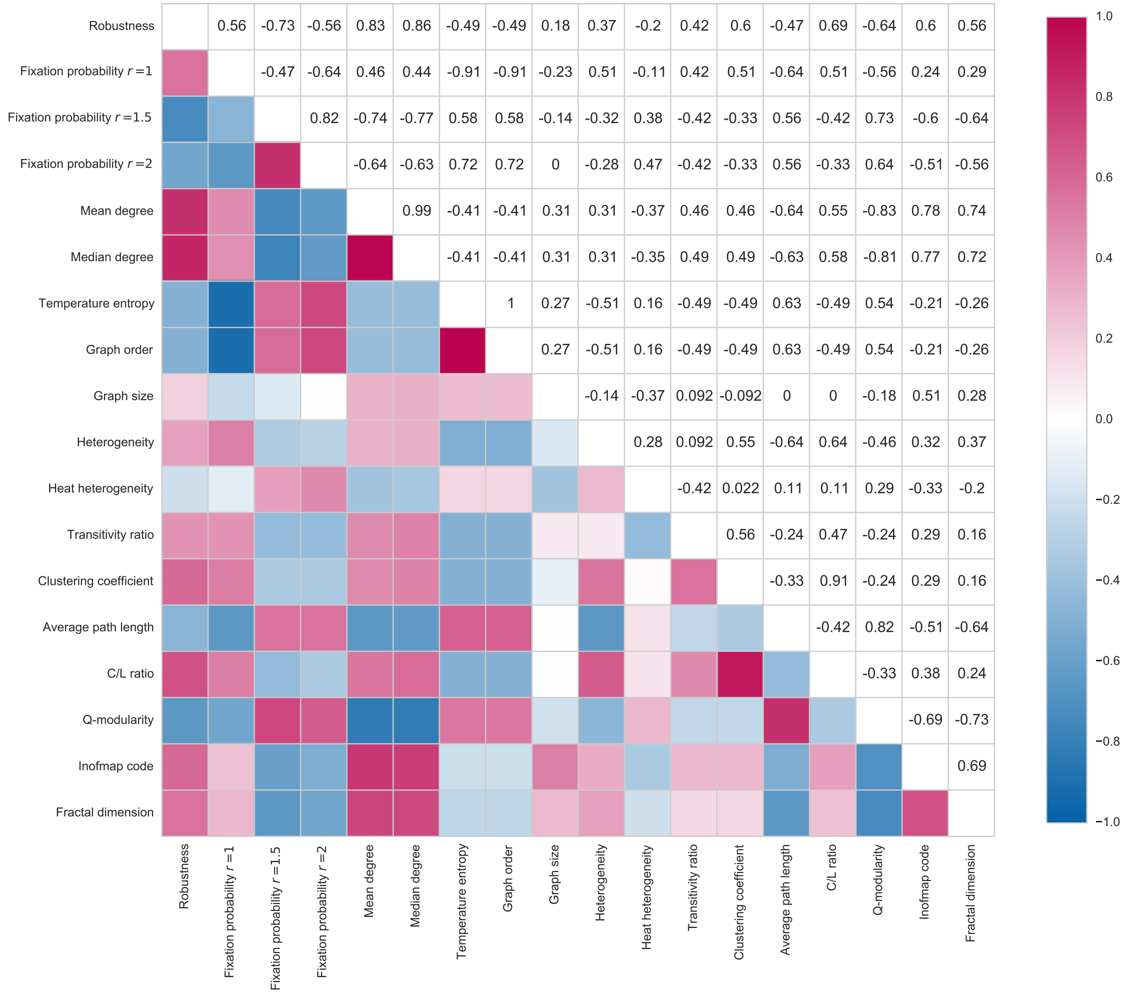

Figure S7. Kendall's rank correlation coefficients between robustness, fixation probabilities, and some statistics, including Q-modularity, I-modularity and fractal dimension.

References

1. Moran, P. A. P. Random processes in genetics. *Proc. Cambridge Philos. Soc.* **54**, 60–71 (1958).
2. Lieberman, E., Hauert, C. & Nowak, M. A. Evolutionary dynamics on graphs. *Nature* **433**, 312–316 (2005).
3. Nowak, M. A. *Evolutionary Dynamics: Exploring the Equations of Life* (Belknap Press of Harvard University Press, 2006).

4. Shakarian, P., Roos, P. & Johnson, A. A review of evolutionary graph theory with applications to game theory. *Biosystems* **107**, 66 – 80 (2012).

5. Taylor, H. M. & Karlin, S. *An introduction to stochastic modeling* (Academic Press Inc., San Diego, CA, 1998), third edn.

6. GlobalNOC. Internet2 Maps & Documentation. `http://noc.net.internet2.edu/i2network/maps-documentation.html`. April 2015.

7. Artzy-Randrup, Y., Fleishman, S. J., Ben-Tal, N. & Stone, L. Comment on ”network motifs: Simple building blocks of complex networks” and ”superfamilies of evolved and designed networks”. *Science* **305**, 1107 (2004). URL `http://www.sciencemag.org/content/305/5687/1107.3.short`. `http://www.sciencemag.org/content/305/5687/1107.3.full.pdf`.

8. Tan, S. & Lu, J. Characterizing the effect of population heterogeneity on evolutionary dynamics on complex networks. *Sci. Rep.* **4**, 2014/05/22/online (2014). URL `http://dx.doi.org/10.1038/srep05034`.

9. Blondel, V. D. The Louvain method for community detection in large networks. `https://perso.uclouvain.be/vincent.blondel/research/louvain.html`. March 2011.

10. Song, C., Gallos, L. K., Havlin, S. & Makse, H. A. How to calculate the fractal dimension of a complex network: the box covering algorithm. *Journal of Statistical Mechanics: Theory and Experiment* **2007**, P03006 (2007). URL `http://stacks.iop.org/1742-5468/2007/i=03/a=P03006`.
